# Evaluation of Few-Shot AI-Generated Feedback on Case Reports in Physical Therapy Education: Mixed Methods Study

**DOI:** 10.2196/85614

**Published:** 2025-12-30

**Authors:** Hisaya Sudo, Yoko Noborimoto, Jun Takahashi

**Affiliations:** 1 The United Graduate School of Education Tokyo Gakugei University Tokyo Japan; 2 Graduate School of Teacher Education Tokyo Gakugei University Tokyo Japan; 3 Faculty of Education Tokyo Gakugei University Tokyo Japan

**Keywords:** large language models, artificial intelligence, AI, generative artificial intelligence, generative AI, Gemini, in-context learning, few-shot setting, formative feedback, algorithm aversion, health profession education, physical therapy education

## Abstract

**Background:**

While artificial intelligence (AI)–generated feedback offers significant potential to overcome constraints on faculty time and resources associated with providing personalized feedback, its perceived usefulness can be undermined by algorithm aversion. In-context learning, particularly the few-shot approach, has emerged as a promising paradigm for enhancing AI performance. However, there is limited research investigating its usefulness, especially in health profession education.

**Objective:**

This study aimed to compare the quality of AI-generated formative feedback from 2 settings, feedback generated in a zero-shot setting (hereafter, “zero-shot feedback”) and feedback generated in a few-shot setting (hereafter, “few-shot feedback”), using a mixed methods approach in Japanese physical therapy education. Additionally, we examined the effect of algorithm aversion on these 2 feedback types.

**Methods:**

A mixed methods study was conducted with 35 fourth-year physical therapy students (mean age 21.4, SD 0.7 years). Zero-shot feedback was created using Gemini 2.5 Pro with default settings, whereas few-shot feedback was generated by providing the same model with 9 teacher-created examples. The participants compared the quality of both feedback types using 3 methods: a direct preference question, the Feedback Perceptions Questionnaire (FPQ), and focus group interviews. Quantitative comparisons of FPQ scores were performed using the Wilcoxon signed rank test. To investigate algorithm aversion, the study examined how student perceptions changed before and after disclosure of the feedback’s identity.

**Results:**

Most students (26/35, 74%) preferred few-shot feedback over zero-shot feedback in terms of overall usefulness, although no significant difference was found between the 2 feedback types for the total FPQ score (*P*=.22). On the specific FPQ scales, few-shot feedback scored significantly higher than zero-shot feedback on fairness across all 3 items: “satisfied” (*P*=.02; *r*=0.407), “fair” (*P*=.04; *r*=0.341), and “justified” (*P*=.02; *r*=0.392). It also scored significantly higher on 1 item of the usefulness scale (“useful”; *P*=.02; *r*=0.401) and 1 item of the willingness scale (“invest a lot of effort”; *P*=.02; *r*=0.394). In contrast, zero-shot feedback scored significantly higher on the affect scale across 2 items: “successful” (*P*=.03; *r*=0.365) and “angry” (*P*=.008; *r*=0.443). Regarding algorithm aversion, evaluations for zero-shot feedback became more negative for 83% (15/18) of the items after identity disclosure, whereas positive perceptions of few-shot feedback were maintained or increased. Qualitative analysis revealed that students valued zero-shot feedback for its encouraging tone, whereas few-shot feedback was appreciated for its contextual understanding and concrete guidance for improvement.

**Conclusions:**

Japanese physical therapy students perceived few-shot feedback more favorably than zero-shot feedback on case reports. This few-shot AI model shows potential to resist algorithm aversion and serves as an effective educational tool to support autonomous writing, facilitate reflection on clinical reasoning, and cultivate advanced thinking skills.

## Introduction

### Background

The integration of generative artificial intelligence (AI) into the health care sector has garnered much attention in recent years, with ongoing debates about its potential and limitations [1-8]. These rapid advancements require a fundamental transformation of various aspects of education, such as learning goals, teachers’ roles, curriculum development, and learner assessment [9]. Health profession education is no exception to this trend [4].

A promising application of this transformative technology is AI-powered formative assessment for case reports, which demonstrates great potential for developing students’ clinical reasoning skills by providing adaptive, real-time, and personalized feedback. Clinical reasoning refers to the essential higher-order cognitive process that clinicians use to reach the right diagnosis and recommend the appropriate therapy [10]. Traditionally, these clinical reasoning skills have been cultivated through the iterative process of discussing real cases, writing case reports, and refining them with feedback from experienced clinicians [10-12]. However, this traditional model faces significant logistical challenges in formal educational settings. For example, providing personalized feedback to each student in an academically diverse cohort is highly demanding on faculty time and resources, making it difficult to implement the frequent cycles of feedback and revision necessary for skill development. The use of AI technology holds significant potential for overcoming these challenges. Although a growing body of research has investigated the effectiveness of AI-powered feedback [13-16], few studies have focused specifically on health profession education.

### Related Work

Formative feedback is information that modifies a learner’s thinking or behavior to improve performance [17]. Recent research has explored the effectiveness of formative feedback generated by large language models (LLMs), such as ChatGPT developed by OpenAI, for enhancing students’ writing skills [18-23]. Specifically, a study by Shi et al [20] found that students receiving feedback from ChatGPT showed significantly higher academic writing performance compared to a control group. Indeed, the overall quality of feedback generated by LLMs has been found to be comparable to that of human experts across several criteria, including linguistic clarity and the use of technical terminology [21]. However, the findings are not uniform. For instance, another study has revealed that peer-generated feedback outperformed that from ChatGPT [22]. Moreover, Escalante et al [23] found that, while there was no significant difference in learning outcomes between ChatGPT and human tutor feedback, each offered distinct advantages. These varied findings suggest that research in this domain is still in its early stages and the debate is ongoing. Consequently, the emerging consensus points toward a hybrid approach that combines the respective strengths of human- and AI-generated feedback rather than debating which is superior [22,24].

Although LLMs possess extensive general knowledge, they often lack depth in specialized fields such as medicine, finance, and law. This limitation often leads to significant inaccuracies and hallucinations when asked questions that require specialized information. Dynamically injecting domain-specific knowledge in real time represents a promising solution to enhance their accuracy and reliability for these specialized tasks [25]. As a viable approach to this challenge, in-context learning has become a significant new paradigm. This approach allows LLMs to make predictions by leveraging a few examples provided within the context [26]. In the in-context learning framework, Brown et al [27] contrasted the zero-shot setting, which relies solely on simple natural language prompts describing the task, with one-shot and few-shot settings, which provide one or several reference examples to help the model understand domain-specific context. Their findings demonstrated that providing just one or a few examples (one-shot and few-shot approaches) resulted in significantly higher performance than the zero-shot approach. A recent study by Rüdian et al [28] evaluated student perceptions of LLM-generated feedback using this few-shot setting in language education. Their approach leveraged 10 pairs of existing student submissions and their corresponding teacher comments to effectively prime the LLM. This priming was further guided by the following prompt: “You are a helpful teacher who provides feedback based on the texts submitted by students. Respond from the first-person perspective.” The results showed that students reported that they could not distinguish LLM-generated feedback with the few-shot setting from feedback created by teachers. This field is still emerging, and further research is required. In particular, there is limited research comparing the quality of formative feedback generated via zero-shot versus few-shot settings. This comparison will provide crucial foundational insights for designing more effective domain-specific AI-generated feedback in education.

For successful integration of LLM-based feedback systems into educational settings, identifying their usefulness and student acceptance is crucial [28]. A key phenomenon in evaluating the usefulness of AI-generated feedback is “algorithm aversion” [29], which refers to the tendency to prefer human advice over that of AI even when the AI’s advice is superior. To investigate the impact of algorithm aversion on AI-generated feedback, Nazaretsky et al [30] examined how students’ perceptions of AI- and human-generated feedback changed before and after revealing the feedback provider’s identity. The results showed that students’ evaluations of the AI-generated feedback became significantly more negative after they were informed of its identity. A promising approach to address algorithm aversion involves integrating human educators’ input into AI-based systems. For example, a study by Zhang et al [31] demonstrated that revealing the source of AI-generated feedback negatively impacted students’ perception of its genuineness, whereas the evaluation of human and AI–coproduced feedback was unaffected by the disclosure of its identity. Our human-AI hybrid methodology differs from the human and AI–coproduced approach described by Zhang et al [31], in which LLMs provide suggestions only when they determine that the teacher-created feedback requires improvement. Given that final human verification of LLM outputs is essential, enhancing the AI’s output itself has the advantages of generating higher-quality feedback and reducing the burden of human review. Therefore, this study contributes to this area by examining whether AI-generated feedback from a few-shot setting demonstrates resistance to algorithm aversion.

To assess the quality of AI-generated feedback on case reports, this study used the Feedback Perceptions Questionnaire (FPQ) [32]. The FPQ is a multidimensional 18-item instrument (shown in Table 1) designed to measure feedback perceptions across 5 scales: fairness, usefulness, acceptance, willingness, and affect. Items were measured on a 100-mm visual analogue scale from “fully disagree” (0) to “fully agree” (100). The 3 items for negative affect (items 16-18) are reverse coded, meaning that a higher score indicates a lower level of that emotion. For example, for the negative affect item “I would feel angry if I received this feedback on my revision,” a check mark for 30 points (indicating low anger) would be reverse coded and calculated as 70 points (100 – 30 = 70) toward the overall affect score. The FPQ is a structurally valid and reliable instrument widely used in higher education research to compare perceptions of different types of feedback [33-35].

**Table 1:** Items of the Feedback Perceptions Questionnaire [32].

Subscale			Items
Fairness			Item 1: “I would be satisfied with this feedback.” Item 2: “I would consider this feedback fair.” Item 3: “I would consider this feedback justified.”
Usefulness			Item 4: “I would consider this feedback useful.” Item 5: “I would consider this feedback helpful.” Item 6: “This feedback would provide me a lot of support.”
Acceptance			Item 7: “I would accept this feedback.” Item 8: “I would dispute this feedback.” Item 9: “I would reject this feedback.”
Willingness			Item 10: “I would be willing to improve my performance.” Item 11: “I would be willing to invest a lot of effort in my revision.” Item 12: “I would be willing to work on further text revision assignments.”
Affect: “I would feel...if I received this feedback on my revision.”			
	Positive	Item 13: satisfied Item 14: confident Item 15: successful	
	Negative	Item 16: offended Item 17: angry Item 18: frustrated	

### Research Objectives

This study aimed to compare the quality of AI-generated formative feedback from 2 different settings, feedback generated in a zero-shot setting (hereafter, “zero-shot feedback”) and feedback generated in a few-shot setting (hereafter, “few-shot feedback”), using a mixed methods approach in Japanese physical therapy education. To examine the effect of algorithm aversion on these 2 feedback types, this study investigated the following research questions: How do physical therapy students evaluate zero-shot and few-shot feedback? How do their evaluations change before and after revealing the identity of the feedback?

The findings offer practical guidelines for the effective integration of generative AI into health profession education.

## Methods

### Participants

An a priori power analysis was conducted using G*Power (version 3.1.9.7) to determine the required sample size for a Wilcoxon signed rank test. The minimum required sample size was calculated to be 74 based on an assumed effect size of 0.3, an α level of .05, and statistical power of 0.80.

A total of 40 fourth-year students from a 4-year physical therapy college in Japan were invited to participate in the study. Of these 40 students, 35 (88%) provided informed consent and were included in the final sample (n=22, 63% male and n=13, 37% female; mean age 21.4, SD 0.7 years). Their mean third-year grade point average was 2.8 (SD 0.5) on a 4-point scale. The final sample size did not reach the 74 estimated by the power analysis. This limitation was due to the exploratory nature of this study, which recruited from a single cohort at a single institution. Consequently, the findings should be interpreted with caution as the reduced statistical power increases the risk of type II error.

### Ethical Considerations

All procedures performed in this study were in accordance with the ethical standards of the Declaration of Helsinki. Written informed consent was obtained from all participants, and a comprehensive verbal and written explanation of the study’s content was provided. This study was approved by the Research Ethics Committee of Tokyo Gakugei University (approval 1022). Participation was voluntary, and no incentives were provided. To protect participant privacy and confidentiality, all data were anonymized, and identifying details were omitted from the analysis and report.

### Procedure

We administered a survey on the use of generative AI tools. This survey included items on (1) past experience with LLMs; (2) the LLM tool they used most frequently; and (3) the frequency of LLM use for 5 specific tasks: academic writing, gathering information, translating, generating new ideas, and proofreading, which were adapted from a large-scale global survey [36]. This frequency was measured on a 5-point Likert scale (1=“never”; 5=“always”).

This study, conducted in July 2025, used a mixed methods approach to compare the quality of zero-shot and few-shot feedback from the perspective of Japanese physical therapy students. This study involved a quantitative evaluation using a questionnaire and a qualitative analysis of semistructured focus group interviews.

The procedure for this study consisted of 4 main phases. First, the 2 types of feedback were generated. Zero-shot feedback was created using the web-based interface of Gemini 2.5 Pro (Google) using the platform’s default settings (eg, default temperature) without any manual parameter adjustments. Few-shot feedback was created using the same model and default settings by providing Gemini 2.5 Pro with 9 examples of prior feedback comments as reference text. These reference comments were written by the first author (16 years of experience as a physical therapist and 5 years of experience as a physical therapy teacher) in May 2025. The comments were for different case reports written by different students. The exact user prompts used to create zero-shot and few-shot feedback, along with an example of prior teacher-created feedback, are provided in Multimedia Appendix 1. Examples of zero-shot and few-shot feedback on a case report are provided in Multimedia Appendix 2. No postprocessing or editing was performed on these 2 outputs.

Second, in a blinded evaluation, participants assessed each type of feedback using the FPQ. After completing the FPQ, participants were asked to indicate which they perceived as more useful overall.

Third, the identity of each feedback type was disclosed to the participants. Participants then re-evaluated the items they wished to change on their initial FPQ responses.

Finally, semistructured focus group interviews were conducted with 26% (9/35) of the participants (3 male and 6 female). They were divided into 3 focus groups of 3 students, each lasting approximately 20 minutes. The interviews explored the rationale behind their perceptions of each feedback type. The interviewees were purposively selected to ensure a mix of students who had received relatively high and low faculty evaluations on their prior case reports.

### Data Analysis

For the quantitative data, descriptive statistics were used to analyze the responses to the direct preference question: “Overall, which feedback comment do you feel is more useful?” The Wilcoxon signed rank test was then used to compare students’ evaluations of zero-shot and few-shot feedback on the median total score on the FPQ and the median scores for each individual FPQ item. Additionally, to address the risk of familywise error from multiple comparisons across the 18 individual FPQ items, adjusted *P* values were calculated using the Bonferroni correction. Following the disclosure of the feedback identity, the number of students whose evaluation became more positive or more negative for each FPQ item was counted. All statistical analyses were performed using Stata/BE (version 19.0; StataCorp LLC), with a significance level set at *P*<.05.

The interviews were audio recorded and transcribed verbatim, with the transcripts serving as the data for analysis. The qualitative analysis followed four main steps: (1) relevant utterances were extracted as meaningful units; (2) each unit was assigned a code; (3) similar codes were inductively grouped into subcategories; and (4) guided by the study’s objective to clarify the characteristics of each feedback type, these subcategories were then organized and integrated into predetermined categories. This analytical process was not strictly linear but recursive, involving movement back and forth between these steps to refine the codes, categories, and themes, consistent with the principles of thematic analysis [37].

We maintained a rigorous audit trail using Google Sheets. The platform’s version history, combined with analytical memos recorded in the document, provided a transparent record of our iterative coding and thematic development process. To ensure the validity of this qualitative analysis, the final codes and categories were determined through discussion and consensus among the 3 authors. Moreover, to verify coding reliability, an external coder (an experienced physical therapy teacher) independently classified 50% (21/42) of the codes into the categories defined by the authors. Interrater reliability was assessed using the Cohen κ. The κ coefficient was 0.504 (95% CI 0.351-0.656), indicating a statistically significant, moderate agreement beyond chance (*Z*=6.47; *P*<.001).

## Results

**Figure 1:**
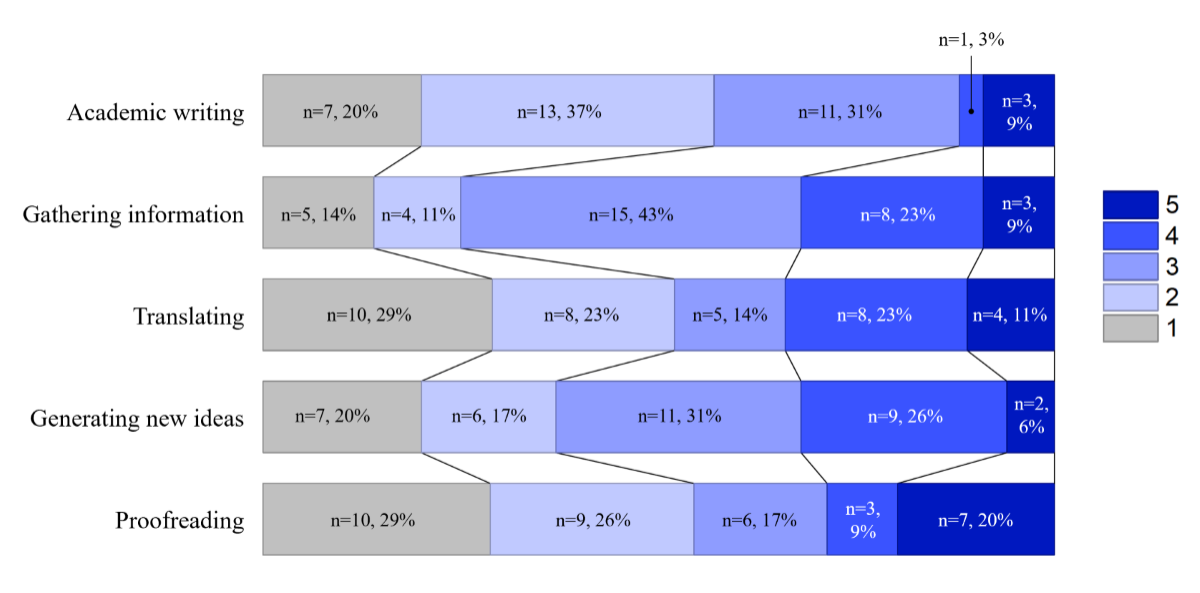
Frequency of large language model use for specific tasks (N=35; 1=“never”; 5=“always”).

All participants reported having prior experience with LLMs. Regarding the most frequently used tool, a clear majority of students reported using ChatGPT (32/35, 91%), followed by Gemini (2/35, 6%) and Microsoft Copilot (1/35, 3%). Figure 1 shows the results for the frequency of LLM use for the 5 specific tasks.

**Figure 2:**
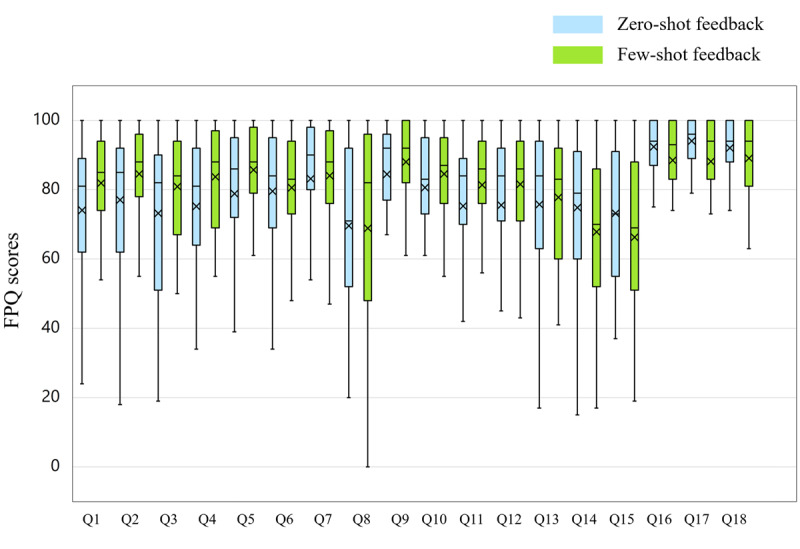
Box-and-whisker plot comparing student perceptions of zero-shot and few-shot feedback across all 18 Feedback Perceptions Questionnaire (FPQ) items. Q: item.

Figure 2 shows the box-and-whisker plot comparing student perceptions of zero-shot and few-shot feedback across all 18 FPQ items. Detailed statistical results of the FPQ scores are provided in Multimedia Appendix 3.

The Cronbach α coefficients indicated acceptable to excellent internal consistency for most scales (Cronbach α≥0.71), with the exception of the acceptance scale for few-shot feedback (Cronbach α=0.58). To further examine the low reliability of this specific scale, additional reliability analyses were conducted for few-shot feedback to examine whether removing an item would improve internal consistency. These analyses indicated that omitting item 8 (retaining items 7 and 9; Cronbach α=0.56), item 9 (retaining items 7 and 8; Cronbach α=0.59), or item 7 (retaining items 8 and 9; Cronbach α=0.31) did not meaningfully increase the reliability beyond the original 3-item scale (Cronbach α=0.58). Therefore, all 3 items were retained, with the understanding that findings related to the acceptance scale must be interpreted with caution.

Regarding the overall usefulness preference, a clear majority of students (26/35, 74%) selected few-shot feedback as more useful than zero-shot feedback, although no significant difference was found between the 2 feedback types for the FPQ score (*P*=.22).

The Wilcoxon signed rank test revealed several statistically significant differences before correction for multiple comparisons. On the specific FPQ scales, few-shot feedback scored significantly higher than zero-shot feedback on fairness across all 3 items: “satisfied” (*P*=.02; *r*=0.407), “fair” (*P*=.04; *r*=0.341), and “justified” (*P*=.02; *r*=0.392). Few-shot feedback also scored significantly higher on 1 item of the usefulness scale (“useful”; *P*=.02; *r*=0.401) and 1 item of the willingness scale (“invest a lot of effort”; *P*=.02; *r*=0.394). In contrast, for the affect scale, zero-shot feedback elicited significantly more positive emotions and less negative emotions. Specifically, students reported feeling significantly more “successful” (*P*=.03; *r*=0.365) and significantly less “angry” (*P*=.008; *r*=0.443) with zero-shot feedback compared to few-shot feedback. However, after applying the Bonferroni correction to address the risk of familywise error from multiple comparisons across the 18 items, none of these differences remained statistically significant (Multimedia Appendix 3 provides detailed adjusted *P* values).

**Figure 3:**
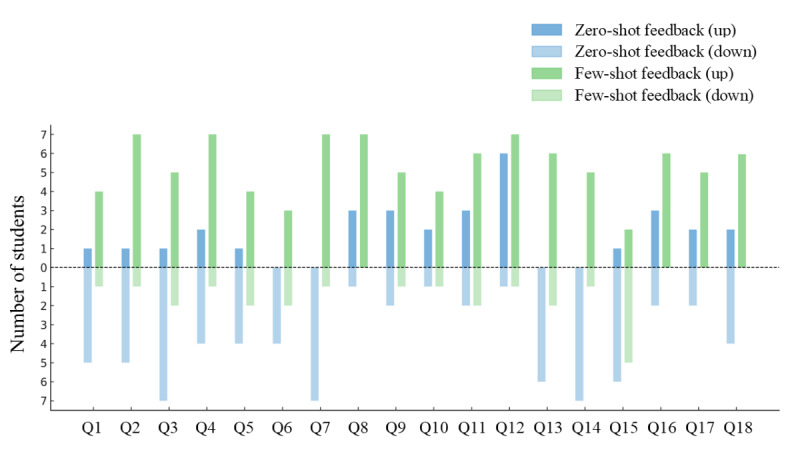
Comparison of the number of students with increased (“up”) or decreased (“down”) evaluation scores for zero-shot and few-shot feedback on each Feedback Perceptions Questionnaire item after the feedback identity was revealed. Q: item.

Figure 3 illustrates the number of students whose evaluations for each of the 18 FPQ items changed after the feedback identities were revealed. Detailed data on the direction of changes in FPQ scores for each item are provided in Multimedia Appendix 4. As this analysis focused on the direction of change (ie, positive or negative) rather than its magnitude, the following description compares the direction in which impressions shifted for each item. After the reveal, evaluations of zero-shot feedback tended to become more negative. For 83% (15/18) of the items, more students lowered their rating for zero-shot feedback than for few-shot feedback. In contrast, evaluations of few-shot feedback tended to become more positive. For all 18 items, a greater number of students increased their rating for few-shot feedback compared to zero-shot feedback. The proportion of students whose evaluations remained completely unchanged was 54% (19/35) for zero-shot feedback and 57% (20/35) for few-shot feedback.

Table 2 presents students’ evaluations of each feedback type through focus group interviews. The qualitative analysis revealed both common and distinct characteristics of zero-shot and few-shot feedback.

**Table 2:** Students’ evaluations of each feedback type.

Category and subcategory		Valence	Speaker ID	Example quotes
**Zero-shot feedback**				
	Praising tone	Positive	D, E, F, G, H, and I	“Zero-shot feedback makes me feel good, so it’s the one I would want to read first to get motivated.” [F]
	Excessive and unrealistic praise	Negative	K and L	“Zero-shot feedback is overpraising—basically nothing but praise. It feels over the top.” [K]
	Enhancement of readability	Positive	J	“The comments on writing structure were helpful.” [J]
	Clarification of vague points	Positive	D, F, H, and J	“Zero-shot feedback is convincing because it points out the areas I am unclear about.” [D]
	Lack of critical comments	Negative	E, G, and H	“Zero-shot feedback gives a lot of praise and is very affirming, but I feel it lacks critical comments, which leaves me feeling unsatisfied and wondering if it’s really good enough.” [E]
**Few-shot feedback**				
	Praising tone	Positive	F	“Few-shot feedback makes me happy because it ends with an encouraging phrase.” [F]
	Justified praise	Positive	L	“I felt few-shot feedback praised the parts it genuinely considered good.” [L]
	Lack of praising tone	Negative	I	“I want feedback to tell me what’s good. If I read only few-shot feedback, I end up thinking, ‘Should I just rebuild the whole thing from scratch?’” [I]
	Enhancement of readability	Positive	D and L	“Few-shot feedback is helpful because it revises the text to make it easier for the reader to understand.” [D]
	Example sentences	Positive	E	“Few-shot feedback is helpful because it gives example sentences, which makes the feedback easier to imagine.” [E]
	Identification of areas for improvement	Positive	I, K, and L	“Few-shot feedback offered comments such as ‘you might also include...’ and ‘it would be good to consider...,’ which made me realize what I was missing. It directly addressed my areas for improvement.” [I]
	Perceived teacher authorship	—^a^	I	“I thought the feedback had been written by the teacher pretending to be AI.” [I]

^a^The valence was not clearly identifiable as positive or negative.

A shared subcategory for both feedback types was “praising tone,” but its quality and degree were perceived differently. For zero-shot feedback, most students (6/9, 67%) found the praise motivating and felt that it made them happy. However, this was also described negatively by some as excessive and unrealistic praise:

Zero-shot feedback is overpraising—basically nothing but praise. It feels over the top.

K

In contrast, few-shot feedback’s praise was perceived as more justified and authentic, although a student noted a lack of praising tone compared to zero-shot feedback:

I felt few-shot feedback praised the parts it genuinely considered good.

L

Regarding the feedback content, zero-shot feedback was positively valued for its clarification of vague points and its enhancement of readability through comments on writing structure. Conversely, a key drawback noted by students was a lack of critical comments, which left them feeling unsatisfied and uncertain about their work’s actual quality.

Few-shot feedback was also praised for its enhancement of readability. Furthermore, students highlighted its unique strengths in providing concrete example sentences, which made the feedback easier to imagine, and its clear identification of areas for improvement. Notably, a student commented on the perceived teacher authorship of few-shot feedback, thinking it had been written by a teacher pretending to be AI.

## Discussion

### Interpretation of the Results

This study aimed to evaluate the quality of few-shot feedback on case reports by comparing it with zero-shot feedback in Japanese physical therapy education using a mixed methods approach. The quantitative results showed that 74% (26/35) of the students preferred few-shot feedback to zero-shot feedback for overall usefulness, although no significant difference was found between the total FPQ scores for the 2 feedback types. The qualitative analysis revealed that zero-shot feedback was favored for its praising tone, whereas few-shot feedback was valued for its concrete guidance for improvement. Furthermore, after the feedback identities were revealed, the positive perception of few-shot feedback was maintained, whereas evaluations for zero-shot feedback tended to become more negative. This suggests that, even though both feedback types were AI generated, the impact of algorithm aversion differs depending on the generation condition.

While no significant difference was observed between the total FPQ scores for zero-shot and few-shot feedback, the Wilcoxon signed rank test on specific FPQ scales demonstrated that few-shot feedback scored significantly higher in fairness, usefulness, and willingness on several key items. Conversely, zero-shot feedback scored significantly higher on the affect scale, eliciting more positive emotions from students. However, after applying the Bonferroni correction for multiple comparisons, none of these differences remained statistically significant. These results must be interpreted with caution given that the study’s statistical power was limited, which increases the risk of type II error. Considering this limitation, coupled with the exploratory nature of this research into the novel area of few-shot feedback, the FPQ items that showed significant differences before correction may indicate potential trends. These items warrant further investigation in future, larger-scale studies, where they might serve to corroborate the findings of the qualitative analysis.

The qualitative analysis revealed that students appreciated the encouraging and praising tone of zero-shot feedback. Regarding few-shot feedback, they valued its more specific and justified praising comments, even if the praise was less frequent. Considering research indicating that LLMs can be more encouraging than humans [38] and that humans tend to focus on areas for improvement rather than praise [39], this suggests that the praising tone of few-shot feedback may have decreased as it imitated these human elements. To ensure a strict comparison between the 2 feedback types in this study, we intentionally made no adjustments to the prompts to control for this praising tone. However, future research should examine how student perceptions change when prompts are adjusted to control for this praising tone as such prompt engineering might lead to few-shot feedback being perceived more favorably by students. Furthermore, our results using Gemini 2.5 Pro may not generalize to other LLMs as outputs can differ significantly between models even when using identical prompts [40-43]. Moreover, as LLMs are updated frequently, future model updates might enable even zero-shot settings to produce more personalized and contextual outputs based on a user’s past data, potentially altering the findings of this study. Given these factors, future research should compare the quality of zero-shot and few-shot feedback across different LLMs.

While students appreciated that zero-shot feedback helped clarify vague points, they noted that it lacked the critical comments necessary for further improvement. In contrast, few-shot feedback was perceived more favorably because it not only pointed out areas for improvement but also offered concrete strategies on how to make those improvements by providing specific textual examples. This ability was interpreted as a form of contextual understanding as the few-shot feedback seemed to infer unstated information from the case report. A study by Pahi et al [38], which explored a collaborative feedback process involving teaching assistants and ChatGPT, revealed that teaching assistants were particularly effective at providing detailed technical comments and identifying conceptual gaps. Similarly, a large-scale study by Henderson et al [39] across 4 Australian universities investigating perceptions of AI and human feedback found that students perceived human feedback as more in depth (nuanced) and contextualized than AI feedback. These findings are consistent with the results of our study and are supported by a report from the Organisation for Economic Co-operation and Development (OECD) [9], which notes that current AI systems still face significant challenges in qualitative reasoning and interpreting unstructured contexts. This suggests that, although no direct human intervention was applied, the human element from the leveraged teacher-created examples may have influenced the few-shot feedback to provide these more conceptual and clinical insights that AI cannot yet fully replicate.

A central finding was the significant shift in student perceptions after disclosure of the feedback’s identity. Feedback effectiveness depends on how students perceive its source (eg, teacher, peer, and parents) regardless of the feedback’s quality [44]. This distinction is crucial when considering algorithm aversion. In our study, zero-shot feedback tended to be re-evaluated negatively after its identity was revealed, which aligns with prior research [31]. This phenomenon likely reflects a lack of trust in the feedback source [30]. In contrast, few-shot feedback showed resistance to algorithm aversion. This suggests that the few-shot setting inherited perceived humanlike qualities and enhanced its trustworthiness [39], thus mitigating the negative effects of algorithm aversion.

### Limitations and Future Directions

While the generalizability of our research is constrained by the limited number of participants and its single-institution context in Japan, the findings hold significant transferability to the broader health sciences education contexts, such as occupational therapy, nursing, and pharmacy. As the challenge of providing high-quality, real-time formative feedback to cultivate students’ clinical reasoning is common to these fields, our few-shot feedback model offers foundational insights for advancing the use of AI in health profession education. To validate the applicability of this model, future work should involve cross-institutional and multidisciplinary studies. Furthermore, comparative trials and longitudinal studies will be necessary to validate the hypothesis that the few-shot feedback can more effectively develop students’ clinical reasoning skills than other feedback types, including human-created feedback.

### Implications

As the few-shot feedback methodology presented in this paper only requires providing an LLM with examples of a teacher’s prior feedback and using a simple prompt, it demands little specialized AI knowledge or skills. The United Nations Educational, Scientific, and Cultural Organization (UNESCO) AI framework [45] states that teachers are expected to acquire skills to adapt or customize AI tools to build human-centered teaching practice. While acquiring such skills is a valuable long-term goal, it can be challenging, particularly for novice teachers. Therefore, the few-shot feedback model offers a highly practical and immediately accessible solution that lowers the barrier to entry for AI integration.

### Conclusions

This study demonstrated that Japanese physical therapy students perceived few-shot feedback more favorably than zero-shot feedback on case reports and that this few-shot feedback model showed potential to mitigate algorithm aversion. This few-shot AI model is expected to serve as an effective support tool that empowers students to autonomously write case reports, reflect on their clinical reasoning processes, and cultivate advanced thinking skills.

## Appendix

Multimedia Appendix 1 Prompts for creating zero-shot and few-shot feedback with an example of prior teacher-created feedback.

Multimedia Appendix 2 Examples of zero-shot and few-shot feedback on a case report.

Multimedia Appendix 3 Detailed statistical results of the Feedback Perceptions Questionnaire scores.

Multimedia Appendix 4 Detailed data on the direction of changes in Feedback Perceptions Questionnaire scores for each item.

## Abbreviations

AI: artificial intelligence
FPQ: Feedback Perceptions Questionnaire
LLM: large language model
OECD: Organisation for Economic Co-operation and Development
UNESCO: United Nations Educational, Scientific, and Cultural Organization
