# Supplementary material for: Evaluation of Few-Shot AI-Generated Feedback on Case Reports in Physical Therapy Education: Mixed Methods Study
Source: JMIR Med Educ. 2025 Dec 30;11:e85614. doi: 10.2196/85614 (PMC12811036; doi:10.2196/85614)
Supplement: Multimedia Appendix 1 [file mededu_v11i1e85614_app1.doc]

**Multimedia Appendix 1.** Prompts for creating zero-shot and few-shot feedback and an example of prior teacher-created feedback.

**Prompt for creating zero-shot feedback**

Please revise the text of a case report written by a physical therapy student based on case information.

Please provide comments as if you were a teacher at a physical therapy college.

**Critique Perspectives**

- Whether the writing is logical.
- Whether the information to support your claims is explained appropriately (e.g., is it consistent with cited information, is any necessary information missing?).
- Whether you can capture a holistic view of the case, not just the local functional impairments.
- Whether the writing is considerate of the reader's ease of understanding.

**Important Notes**

- Please provide comments alongside corresponding quotes from the original text.
- Please use appropriate line breaks in your comments on the original text to ensure clarity.
- Please format your comments to be easily identifiable, such as with [Comment].
- Please use underlining or bolding to emphasize important comments.

=== Text for Revision ===

**Prompt for creating few-shot feedback**

Please refer to the comments in the provided examples and comment as if you were the teacher who wrote them.

**Important Notes**

- Please provide comments alongside corresponding quotes from the original text.
- Please use appropriate line breaks in your comments on the original text to ensure clarity.
- Please format your comments to be easily identifiable, such as with [Comment].
- Please use underlining or bolding to emphasize important comments.

=== Text for Revision ===

**An example of prior teacher-created feedback**

**Feedback Comments on a Case Report of Anterior Cruciate Ligament (ACL) Injury**

**Comment 1**: First, it would be better to describe the history in more detail. It is important to enable the reader to form a specific image of this case here. Information such as the mechanism of injury (contact or non-contact), surgical procedure, presence of meniscal injury, dates of injury and surgery, current postoperative month, date of mobilization (if applicable), subsequent progress, and the player's position is necessary.

**Comment 2:** It would be easier to read if you described the chief complaint specifically. For example, how does the patient express their anxiety or fear?

**Comment 3:** I also want to know to what extent the patient can perform jogging and dashing (e.g., at what percentage of full effort).

**Comment 4:** The phrase "lose balance" feels somewhat colloquial. You should be conscious of expanding your vocabulary and using other expressions, such as "unsteadiness was observed" or stating the objective fact like "the holding time was [X] seconds."

**Comment 5:** The object "center of gravity" is missing. If you include descriptions like "no unsteadiness was observed" or "maintained stably for more than [X] seconds," it is self-evident that the center of gravity is within the base of support, so you might not need to state it explicitly.

**Comment 6:** The expression "lateral oscillation of the knee joint increased" is difficult to visualize, so please use more appropriate kinematic terminology. Likely, "the knee joint goes into a valgus position" would be the appropriate expression here.

**Comment 7:** The expression feels ambiguous. Ambiguity increases the likelihood that the reader's understanding of the movement will vary.

**Comment 8:** If you are claiming that the knee valgus moment tends to increase just because the passive ROM angle is large, please provide the rationale. Without it, this could be dismissed as mere conjecture.

**Comment 9:** There are "external" and "internal" moments. In this case, I believe the correct expression would be "the external knee valgus moment tends to increase." Please review your knowledge in this area.

**Comment 10:** It would be more persuasive if you could state the factual observation of whether "hip internal rotation movement occurred" during the cutting motion.

**Comment 11:** You consistently state that poor alignment is the cause of increased anxiety during cutting motions, but the basis for this claim is weak. If asked, "Aren't there players who can cut without anxiety even with poor alignment?", it is important to be able to explain other factors. For example, I have questions regarding other elements: Is there sufficient basic muscle strength (Hamstrings MMT is noted as 3)? Is the surgical method (STG) having an influence? If knee extension ROM is limited and extension strength has not returned, how much are those factors likely affecting the anxiety?
