# Supplementary material for: Evaluation of Few-Shot AI-Generated Feedback on Case Reports in Physical Therapy Education: Mixed Methods Study
Source: JMIR Med Educ. 2025 Dec 30;11:e85614. doi: 10.2196/85614 (PMC12811036; doi:10.2196/85614)
Supplement: Multimedia Appendix 2 [file mededu_v11i1e85614_app2.doc]

**Multimedia Appendix 2.** Examples of zero-shot and few-shot feedback on a case report.

**An example of zero-shot feedback (Student F)**

**[Original Text]**This patient sustained an ACL injury to the right knee during basketball practice and underwent reconstruction surgery. Rehabilitation was initiated with the goal of returning to sport, with an initial evaluation at 4 weeks post-surgery and a final evaluation at 8 weeks post-surgery. Based on the evaluations and following protocol, the long-term goal (LTG) was set as the initiation of jogging at 3 months post-surgery. The criteria for initiating jogging were no limitation in knee joint range of motion, achievement of the single-leg stand-up test (40 cm), and acquisition of movement stability during the Single-Leg Squat (SLSQ).

**[Comment]**As an introduction, the patient’s background, evaluation timing, long-term goal (LTG), and its achievement criteria are concisely summarized and very easy to understand. If I were to add one thing, the criterion "acquisition of movement stability during the SLSQ" would improve the evaluation’s reproducibility if it could be indicated with more objective metrics. For example, striving for specific descriptions that anyone can judge similarly, such as "no pelvic tilt or knee joint instability (Knee-in/out) observed during SLSQ," would make for an even stronger report.

**[Original Text]**
Among the criteria set for initiating jogging (the LTG), physical therapy was implemented targeting the acquisition of SLSQ movement stability, which was not achieved at the initial evaluation. As a result, movement stability was acquired by the final evaluation.

[**Comment]**
This single sentence clearly indicates that the central theme discussed in this case study is the "improvement of SLSQ stability." The thesis is clear, making it a good introduction that helps the reader easily understand the content that follows.

**[Original Text]**In the SLSQ at the initial evaluation, instability of the right knee joint was observed, and muscle weakness of the surrounding knee joint muscles, as well as a decline in knee joint position sense, were identified as problems. The SLSQ requires co-contraction of the quadriceps and hamstrings¹⁾, and improving the strength of both muscles is necessary to acquire stability in single-leg movements. Furthermore, since knee joint position sense contributes to appropriate postural control and knee joint stability²⁾, it was hypothesized that the knee instability was likely related not only to simple muscle weakness around the knee joint but also to proprioceptive dysfunction, including a decline in joint position sense.

**[Comment]**It is excellent that you were able to extract the two functional impairments, "muscle strength" and "proprioception," from the SLSQ phenomenon. The accurate use of citations to support your own reasoning is also highly commendable. If you describe what kind of instability the "knee joint instability" specifically was (e.g., medial/lateral instability, or anterior/posterior instability like knee buckling), the clinical picture will be conveyed more vividly. Additionally, adding a sentence that directly links the information from citation 1) "co-contraction is necessary" and the "instability" observed in the evaluation, such as, "It was thought that this co-contraction became inefficient due to muscle weakness, leading to knee instability," would further strengthen the logical connection.

**[Original Text]**
The reasons for identifying these problems were that the initial evaluation confirmed left-right differences in thigh circumference, instability of the right knee joint during the single-leg stand-up test, muscle weakness in the hamstring MMT, and a decline in right knee joint position sense.

**[Comment]**
You have specifically shown the evaluation items that form the basis for the problems mentioned in the previous paragraph, clearly demonstrating a logical thought process. As a structural suggestion, this content could also be integrated into the previous paragraph. For example, writing "...were identified as problems. As supporting evidence, the initial evaluation confirmed left-right differences in thigh circumference and hamstring MMT weakness. Furthermore, ..." would group the problems and their supporting evaluation results together, making the text flow more smoothly.

**[Original Text]**
Regarding the left-right difference in thigh circumference, it was thought to be largely due to arthrogenic muscle inhibition (AMI) caused by joint effusion or muscle atrophy from decreased postoperative activity. The hamstring weakness was considered to be significantly influenced by the harvesting of the semitendinosus tendon during the reconstruction surgery. Additionally, the decline in knee joint position sense was thought to be caused by the loss of mechanoreceptors within the ligament due to the ACL injury and muscle weakness of the surrounding knee joint muscles²⁾.

**[Comment]**You have deeply analyzed the evaluation results based on pathological and medical knowledge, such as arthrogenic muscle inhibition (AMI), loss of mechanoreceptors, and the influence of the surgical procedure. This attitude of not just describing phenomena but trying to interpret the underlying mechanisms is extremely important for a clinician. Citation 2) shows the relationship between knee torque and proprioception; using a more specialized reference (such as a review on ACL and mechanoreceptors) as a direct citation for "loss of mechanoreceptors due to ACL injury" would be more persuasive, but the intent to apply knowledge and analyze is well conveyed.

**[Original Text]**
To address these problems, various treatment programs were implemented, such as blood flow restriction (BFR) training, squats, leg presses, leg curls, and training using a mirror for visual feedback. As a result, the left-right difference in thigh circumference improved from a maximum of 2 cm at the initial evaluation to 0.5 cm, and the hamstring MMT improved from 4 to 5, indicating clear muscle hypertrophy and strength gains. Furthermore, regarding knee joint position sense, reproduction errors were seen in 2 out of 5 trials at the initial evaluation, but no errors were observed at the final evaluation, confirming improvement.

**[Comment]**The interventions and the resulting improved evaluation results (outcomes) are shown with specific numbers, objectively demonstrating the effectiveness of the interventions. To make this a higher-quality analysis, clearly describe the purpose: "Why did you choose these treatment programs?" For example, explicitly stating how each intervention corresponds to which problem, such as "BFR training and leg presses were implemented to improve muscle strength, and squats using a mirror were performed to improve both muscle strength and proprioceptive function," will clearly convey your thought process.

**[Original Text]**
The treatment program was progressively modified to include training with weights and movements specific to basketball. In this process, when performing a task of higher intensity than the SLSQ, pronation of the right subtalar joint was observed. Subtalar joint pronation affects the ascending kinetic chain, such as tibial internal rotation and knee joint valgus³⁾. Many non-contact ACL injuries occur with added tibial internal rotation and knee joint valgus⁴⁾; therefore, the ascending kinetic chain induced by subtalar joint pronation could be a factor in forming the injury position. Consequently, training using a balance board (Blackboard) was implemented to enhance sensory input to the foot, which reduced the subtalar joint pronation and improved foot instability. From these results, it was determined that continuing sensory input to the foot is necessary. Furthermore, training of unaffected areas such as trunk and hip surrounding muscles, which had been actively conducted from the initial phase, will also be continued.

**[Comment]**
This paragraph is the most outstanding part of this report. You did not stop at solving the local problem of the knee joint but also noticed a foot problem that emerged during higher-intensity movements, analyzing it from the perspective of the kinetic chain. Furthermore, linking this to the ACL injury mechanism and discussing the necessity of intervention from the important viewpoint of re-injury prevention is precisely the kind of thinking required of a physical therapist. If you add specifics about what the "task of higher intensity than the SLSQ" was (e.g., landing from a light jump, side-steps), the situation will be better understood. Regarding the necessity of continuing training of unaffected areas, mentioning the role of trunk and hip function within the kinetic chain (e.g., reducing the load on the knees and feet) would add even more persuasive power.

**[Original Text]**
Through this intervention, the patient has been acquiring the physical functions necessary for jogging, but since the doctor's clearance is not until 3 months post-surgery, jogging was not initiated within the clinical training period. With continued intervention for another month, it is considered fully possible to start jogging at the 3-month post-surgery mark. After achieving the LTG of initiating jogging, looking ahead to returning to sport regarding running movements, the operational speed will increase, transitioning to higher-intensity and more explosive movements such as running and sprinting. Therefore, not only basic muscle strength and range of motion, but also movement accuracy and dynamic postural control ability via proprioception will become even more crucial. Based on these points, it is expected that implementing interventions that can handle high-intensity and various movements will lead to a return to sport at a higher performance level.

**[Comment]**
You have described the limitations of the clinical training, the future outlook, and the prospects for rehabilitation after starting jogging from a long-term and realistic perspective, making it a very appropriate conclusion to the case study. Looking ahead to the final goal of returning to sport, you clearly indicate the significance of the functions acquired now and what needs to be done next. By providing one or two specific examples of "interventions that can handle high-intensity and various movements" (e.g., plyometrics, cutting drills), your recommendations will become more concrete. Overall, this was a logical and multifaceted, wonderful analysis. Please continue learning at this pace.

**An example of few-shot feedback (Student F)**

Overall, you have demonstrated a logical thought process, and the content is excellent. On top of that, I will provide some comments from the perspective of further deepening your thinking and enhancing your clinical practical abilities.

**[Original Text]**Among the criteria set for initiating jogging (the LTG), physical therapy was implemented targeting the acquisition of SLSQ movement stability, which was not achieved at the initial evaluation. As a result, movement stability was acquired by the final evaluation.

**[Comment]**You clearly identified the challenge for achieving the LTG and described your approach and results at the beginning, which makes for a very understandable introduction to your discussion. However, the expression "acquisition of movement stability" is somewhat abstract. It would convey the effects of your intervention more clearly if you could describe more specifically "what kind of instability (e.g., knee valgus, lateral trunk tilt)" was seen "to what extent," and "how" it improved by the final evaluation.

**[Original Text]**The SLSQ requires co-contraction of the quadriceps and hamstrings¹⁾, and improving the strength of both muscles is necessary to acquire stability in single-leg movements. Furthermore, since knee joint position sense contributes to appropriate postural control and knee joint stability²⁾, it was hypothesized that the knee instability was likely related not only to simple muscle weakness around the knee joint but also to proprioceptive dysfunction, including a decline in joint position sense.

**[Comment]**It is excellent that you analyzed the functions necessary for SLSQ based on literature and listed multiple factors (muscle strength, proprioception). On the other hand, was there an evaluation finding (a fact) in this case that "co-contraction was not being performed well"? While objective assessment is difficult without electromyography, your hypothesis would be more persuasive if it could be supported by a clinical evaluation, such as confirming muscle contraction timing via palpation. Always be conscious of strongly linking "knowledge (literature)" with "the patient's facts (evaluation results)."

**[Original Text]**To address these problems, various treatment programs were implemented, such as blood flow restriction (BFR) training, squats, leg presses, leg curls, and training using a mirror for visual feedback.

**[Comment]**Please explain the rationale for choosing these "various" treatment programs more specifically. For example: "Squats and leg presses were used for muscle weakness, and leg curls were implemented specifically to strengthen the hamstrings from which the tendon was harvested. Additionally, BFR training, which can expect hypertrophic effects even at low loads, was chosen considering the possibility of remaining arthrogenic muscle inhibition. For the decline in knee joint position sense, training in front of a mirror using visual feedback was deemed effective and implemented." Clarifying which problem each program addresses and through what mechanism will enhance the logic of your treatment program design.

**[Original Text]**In this process, when performing a task of higher intensity than the SLSQ, pronation of the right subtalar joint was observed. ... (omitted) ... Consequently, training using a balance board (Blackboard) was implemented to enhance sensory input to the foot, which reduced the subtalar joint pronation and improved foot instability.

**[Comment]**Discovering a new problem during the intervention process, analyzing it, and modifying/adding to your approach is a critical thought process for a clinician. This is an excellent observation. One question: did you evaluate the foot's alignment and function at the initial evaluation stage? If that evaluation was lacking, you could write a "reflection" to apply this experience next time, such as, "At the initial evaluation, I focused on the knee joint area, and the foot evaluation was insufficient. I believe it was necessary to conduct a systemic evaluation, including the foot, from the beginning, considering the kinetic chain." This would make the report show even deeper learning.

**[Original Text]**The treatment program was progressively modified to include training with weights and movements specific to basketball. ... (omitted) ... it was determined that continuing sensory input to the foot is necessary. Furthermore, training of unaffected areas such as trunk and hip surrounding muscles, which had been actively conducted from the initial phase, will also be continued.

**[Comment]**Analyzing the future outlook in detail is very good. In particular, it is excellent that you continue to recognize the importance of training unaffected areas. Your analysis would become more practical and high-quality if you could also briefly touch upon specific points of caution when transitioning to high-intensity training (e.g., evaluating asymmetry for re-injury risk management, qualitative assessment of jumping and cutting movements, psychological support, etc.). Overall, this was a good report that conveyed your sincere approach to clinical practice. Please keep up the good work.
